# Mediation model with a categorical exposure and a censored mediator with application to a genetic study

**DOI:** 10.1371/journal.pone.0257628

**Published:** 2021-10-12

**Authors:** Jian Wang, Jing Ning, Sanjay Shete

**Affiliations:** 1 Department of Biostatistics, The University of Texas MD Anderson Cancer Center, Houston, Texas, United States of America; 2 Department of Epidemiology, The University of Texas MD Anderson Cancer Center, Houston, Texas, United States of America; Universite de Montreal, CANADA

## Abstract

Mediation analysis is a statistical method for evaluating the direct and indirect effects of an exposure on an outcome in the presence of a mediator. Mediation models have been widely used to determine direct and indirect contributions of genetic variants in clinical phenotypes. In genetic studies, the additive genetic model is the most commonly used model because it can detect effects from either recessive or dominant models (or any model in between). However, the existing approaches for mediation model cannot be directly applied when the genetic model is additive (e.g. the most commonly used model for SNPs) or categorical (e.g. polymorphic loci), and thus modification to measures of indirect and direct effects is warranted. In this study, we proposed overall measures of indirect, direct, and total effects for a mediation model with a categorical exposure and a censored mediator, which accounts for the frequency of different values of the categorical exposure. The proposed approach provides the overall contribution of the categorical exposure to the outcome variable. We assessed the empirical performance of the proposed overall measures via simulation studies and applied the measures to evaluate the mediating effect of a women’s age at menopause on the association between genetic variants and type 2 diabetes.

## Introduction

Mediation analysis is a statistical method used to evaluate the direct and indirect effects of an exposure on an outcome in the presence of a mediator. Mediation models have been widely used to determine direct and indirect contributions of genetic variants in clinical phenotypes, such as contribution of *CHRNA3-A5* genes in lung cancer [1–7]. In many studies, one encounters right- or left-censored data instead of complete data. Approaches to assess mediation when the outcome variable is censored have been widely studied [8–15]. However, the mediator itself can also be a censored variable. For instance, genes may affect the age at which a person stops smoking, a variable that is censored for current smokers and has been associated with lung cancer–associated mortality [16]. Few studies have considered mediation models with a censored mediator. Wang and Shete [17] used a multiple imputation approach along with the accelerated failure time (AFT) model to address a censored nature of a mediator when outcome was a continuous variable, and this approach yielded accurate estimations for the coefficients of different paths, the indirect effect (*IE*), and proportion of the total effect mediated (*PM*) by the mediator. Wang et al. [18] further extended the mediation approach with a censored mediator for studies with binary outcomes (e.g., case-control studies), based on the semiparametric AFT model with an unspecified error distribution combined with a pseudo-likelihood function, which was shown to be efficient yet robust.

In genetic association studies of complex diseases—including our motivating study of the association between single nucleotide polymorphisms (SNPs), woman’s age at menopause, and type 2 diabetes—because often there is no concrete evidence of the genetic mode of inheritance, one usually uses three classic genotypic models: the additive, dominant, and recessive genetic models [19]. For example, consider a SNP with two alleles *R* and *r*, and let *R* be the risk allele and *r* be the normal allele. The additive genetic model is defined using a categorical random variable, *X* = (0, 1, 2), to denote the three genotypes (*rr*, *Rr*, *RR*), assuming that the disease risk depends on the dose of the risk allele *R*. When the dominant or recessive genetic model is assumed, we use a binary variable, *X* = (0, 1, 1) or *X* = (0, 0, 1), respectively, to denote the three genotypes (*rr*, *Rr*, *RR*). The additive genetic model is the most commonly used model because typically the mode of action of susceptibility SNPs is unknown and the additive model can detect effects from either recessive or dominant models, or any model in between [20–22]. In addition to SNPs, the highly polymorphic loci, such as human leukocyte antigen (HLA) genes, can also be involved in the mediation model as an exposure. Such genes have many different alleles, resulting in many different genotypes (i.e., more than three genotypes found in di-allelic SNPs). The previous approaches by Wang and Shete [17] and Wang et al. [18] assumed that the exposure is either continuous (e.g., gene expression) or binary (e.g., dominant or recessive mode of inheritance for a genetic variant). These methods therefore cannot be directly applied when the genetic model is additive (e.g. the most commonly used model for SNPs) or categorical (e.g. polymorphic loci), and thus, modification to measures of indirect and direct effects is warranted for these general scenarios. Therefore, we extended our approach to the scenario in which the exposure is a categorical variable which can be applied to the model where the mediator is subject to censoring.

In particular, we proposed the measures for calculating the overall *IE*, direct effect (*DE*), and total effect (*TE*) in such a mediation model, where we first assessed the *IE*, *DE*, and *TE* for each category of the exposure and then calculated the *IE*, *DE*, and *TE*, weighted by the frequency of each category (e.g., frequency of each genotype), to estimate overall effect (*IE*, *DE*, and *TE*) of the exposure on the outcome variable in the presence of a censored mediator. The proposed measures provide the overall contribution (indirect, direct and total) of the categorical exposure to the outcome variable, instead of the relative contribution comparing one category to another in previous studies [23,24]. The proposed measures are general and valid regardless of whether the outcome variable and mediator are continuous, binary, or censored.

**Fig 1:**
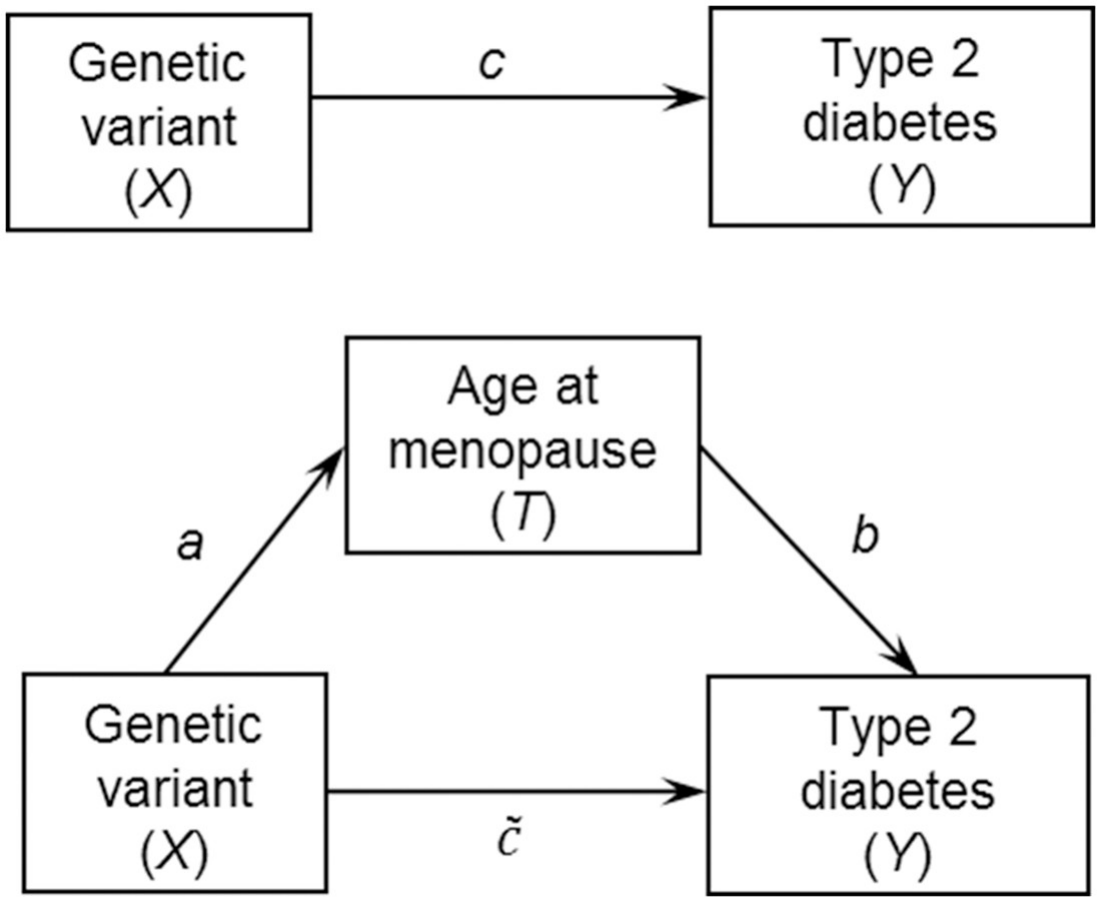
Conceptual model for the study of the mediating effect of a women’s age at menopause on the association between genetic variants and type 2 diabetes risk. Nodes represent the variables being analyzed in the mediation model, including genetic variant (i.e., exposure), a women’s age at menopause (i.e., mediator) and type 2 diabetes (i.e., outcome). A direct edge implies a potential direct causal effect. A pathway from one variable (genetic variant) to another (type 2 diabetes) implies a potential causal relationship through the mediator on the path (age at menopause).

We applied the proposed overall measures of *IE*, *DE*, and *TE* to the motivating study of the mediating effect of a woman’s age at menopause on the effect of SNPs on risk of type 2 diabetes. Type 2 diabetes is a complex disease characterized by interplay between genetic and environmental factors [25–30]. Previous studies using epidemiologic data or genetic epidemiologic data have suggested an association between a woman’s age at menopause and type 2 diabetes [31–33] as well as an association between several SNPs and a woman’s age at menopause [34,35]. Therefore, we hypothesized that there are potential dual pathways between SNPs and type 2 diabetes, one via a direct effect and the other indirectly through a women’s age at menopause, which is hypothesized as a mediator for this association and is a censored variable because not all women have had gone through menopause (Fig 1).

In Methods section, we introduce the notations; mediation models; definitions of the overall *IE*, *DE*, and *TE*; and the associated estimation approaches [18]. We assess the empirical performance of the proposed overall *IE*, *DE* and *TE* via simulation studies in the Simulation section, conduct a data analysis in the section of Application to the motivation study, and provide a discussion in the Discussion section.

## Methods

We first review the approach by Hayes and Preacher [23], which is a widely used mediation model in which the exposure has multiple categories, and point out its limitations in the context of our motivating study. In their approach, Hayes and Preacher proposed to code the multi-categorical exposure using different coding strategies, including dummy coding, contrast coding etc., depending on the research interest. For example, when using the dummy coding, if the multi-categorical exposure *X* has *k* groups, one can create *k*-1 dummy variables *X*_*i*_, *i* = 1, … *k*-1, with *X*_*i*_ = 1 if the subject is in group *i* and *X*_*i*_ = 0 otherwise, where one group is considered the reference group in the analysis. In this way, the approach creates multiple binary exposure variables in one mediation model.

As assumed in the Hayes and Preacher model, when outcome *Y* and mediator *T* are continuous, the direct and indirect effects of *X* on *Y* are estimated using the dummy-coded multiple exposure variables, mediator, and outcome using following linear regressions:

$$\begin{array}{ll} T= & a_{0}+a_{1}X_{1}+\cdots +a_{k-1}X_{k-1}+\varepsilon_{1}\\ Y= & b_{0}+bT+{\tilde{c}}_{1}X_{1}+\cdots +{\tilde{c}}_{k-1}X_{k-1}+\varepsilon_{2}\\ Y= & c_{0}+c_{1}X_{1}+\cdots +c_{k-1}X_{k-1}+\varepsilon_{3}, \end{array}$$

where *a*_*i*_ represents the path from the dummy-coded exposure *X*_*i*_ to the mediator *T*, ${\tilde{c}}_{i}$ represents the path from *X*_*i*_ to the outcome *Y* conditional on the mediator *T*; *c*_*i*_ represents the relative total effect of *X*_*i*_ on the outcome *Y*; *i* = 1, … *k*-1; and *b* represents the effect of the mediator *T* on the outcome *Y*. To assess the mediating effects, Hayes and Preacher adopted the terms relative *IE*, relative *DE*, and relative *TE*, respectively, to refer to *a*_*i*_*b*, ${\tilde{c}}_{i}$, and $c_{i}=a_{i}b+{\tilde{c}}_{i}$; and the effects were calculated for each of the binary exposure variables recoded from the original exposure. (see details in [23]).

The approach proposed by Hayes and Preacher has advantages over other approaches such as aggregating groups or discarding to construct a dichotomous exposure, but it still has certain limitations. Since the relative *IE* is calculated for each created binary exposure separately, it cannot provide the overall mediating effect of the mediator on the relation between the exposure and outcome variable. Also, if the exposure has many categories, the number of possible tests to be conducted can be large. In this case, multiple correction tests reduce the power of the test for the mediating effect. Importantly, the approach proposed by Hayes and Preacher [23] assumes that both outcome and mediator are continuous and normally distributed, which allows one to estimate the relative *TE* as the summation of relative *IE* and relative *DE*. Therefore, the approach cannot be directly applied in many practical situations, such as when the mediator is a non-normal distributed variable subject to censoring and the outcome is binary. More recent work has been conducted to develop approaches for the analysis of treatment/exposure with multiple categories [24,36–38]. However, these approaches compare one category to another of the treatment/exposure without providing overall direct and indirect effects of the categorical exposure variable on the outcome variable. Furthermore, the direct application of these methods to a mediation model with a censored mediator and a binary outcome is not straightforward. For instance, the approaches proposed by Samoilenko et al. focused on continuous mediator and outcome [24].

To address these limitations, we proposed an approach to assess the overall mediating effect of a mediation model in which the exposure is categorical, and the mediator is subject to censoring. In the next sections, we propose definitions for the overall measures for *IE*, *DE*, and *TE* by extending the approach proposed in Wang et al. [18]. We focused on this approach because, compared to other existing approaches, it employed the semiparametric AFT model, which does not require a parametric distribution assumption on the mediators, and the pseudo-likelihood function, which is more flexible to be extended to different outcome variables (e.g., continuous outcome). However, as mentioned above, the proposed overall measures of *IE*, *DE* and *TE* are general and valid regardless of whether the outcome variable and mediator are continuous, binary, or censored or the approaches used.

### *IE* for a mediation model with a categorical exposure and a continuous outcome

**Fig 2:**
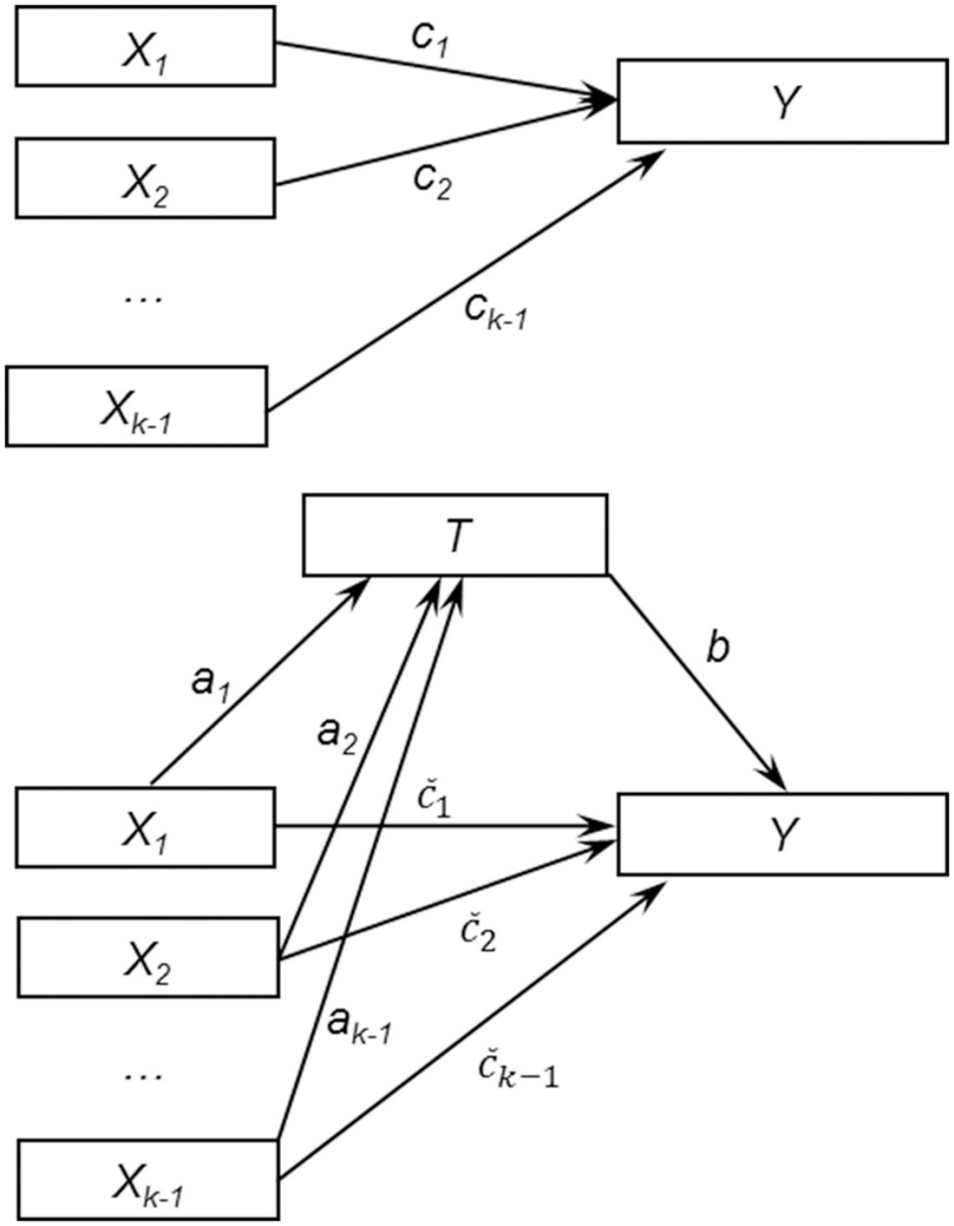
Mediation model with *k-1* dummy variables created for the categorical exposure with *k* groups. Nodes represent the variables being analyzed in the mediation model, including the *k*-1 dummy-coded exposure variables, *X*_1_, *X*_2_, …, *X*_*k*−1_, the mediator *T* and the outcome *Y*. A direct edge implies a potential direct causal effect. A pathway from one variable (e.g., *X*_1_) to another (*Y*) implies a potential causal relationship through the mediator on the path (*T*).

We first present methodology for the scenario when outcome variable is continuous. Let *X* be the categorical exposure, *T* be the mediator subject to right censoring, *Y* be the continuous outcome variable and *Z* be the other covariates involved in a mediation model. For the mediator, given an individual *i*, *i* = 1, …, *n*, we observe *m*_*i*_ = min(*t*_*i*_, *c*_*i*_) and *δ*_*i*_ = *I*(*t*_*i*_ ≤ *c*_*i*_), where *c*_*i*_ is the right-censored time and *δ*_*i*_ is the indicator for censored (*δ*_*i*_ = 0) or observed (*δ*_*i*_ = 1). For the categorical exposure, we utilize the dummy coding as suggested in Hayes and Preacher. If the categorical exposure *X* has *k* categories, we create *k*-1 dummy variables, *X*_*j*_, *j* = 1, … *k*-1, with one of the categories as the reference (Fig 2), where *X*_*j*_ = 1 if *X* = *d*_*j*_, and *X*_*j*_ = 0 if *X* ≠ *d*_*j*_. That is, *X* = *d*_*j*_ means (*X*_1_ = 0, … *X*_*j*_ = 1, … *X*_*k*−1_ = 0). For the reference category, *X* = *d*_0_ is equivalent to (*X*_1_ = 0, … *X*_*j*_ = 0, … *X*_*k*−1_ = 0). For example, in our motivation study, the exposure is coded as an additive genetic model with three categories (0, 1, 2) denoting the three genotypes (*rr*, *Rr*, *RR*). Here *k* = 3 and we create two dummy variables, *X*_1_ and *X*_2_, using the genotype *rr* (i.e., 0) as the reference group. Specifically, *X*_1_ = 1 when *X* = 1 (genotype *Rr*) and *X*_2_ = 1 when *X* = 2 (genotype *RR*).

The relationships among the variables can be specified using the linear regression model (i.e., association of exposure and mediator with outcome) and AFT model (i.e., association of exposure with mediator) as follows:

$$y_{i}=b_{0}+bt_{i}+\sum_{j=1}^{k-1}{\tilde{c}}_{j}x_{ji}+{\gamma^{T}z_{i}+\varepsilon }_{yi},$$

and

$$log\left(t_{i}\right)=a_{0}+\sum_{j=1}^{k-1}a_{j}x_{ji}+{\tilde{\gamma }}^{T}z_{i}+\varepsilon_{ti}=A_{i}^{T}\theta +\varepsilon_{ti},$$

where *b*_*0*_, *b*, ${\tilde{c}}_{j}$, *j* = 1, …, *k* − 1, *γ*, and $\theta ={\left(a_{0},a_{1},\ldots ,a_{k-1},{\tilde{\gamma }}^{T}\right)}^{T}$ are the regression coefficients; $A_{i}={\left(1,x_{1i},\ldots ,x_{(k-1)i},z_{i}^{T}\right)}^{T}$; *ε*_*yi*_ ~ Normal(0, *σ*^2^); and *ε*_*ti*_ represents the independently and identically distributed random errors with mean zero and an unspecified distribution. Particularly, the coefficients *a*_*j*_, *j* = 1, …, *k* − 1, correspond to the paths from *k*-1 dummy variables created for the original exposure, *X*_1_, *X*_2_, …, *X*_*k*−1_, to the mediator *T*; the coefficients ${\tilde{c}}_{j}$, *j* = 1, …, *k* − 1, correspond to the paths from *k*-1 dummy-coded exposure variables to the outcome *Y*; and *b* corresponds to the path from the mediator *T* to the outcome *Y*. In the presence of right-censoring, given a continuous outcome, the likelihood function for the observed data (*y*_*i*_, *m*_*i*_, *δ*_*i*_, *x*_*1i*_, …, *x*_*(k-1)i*_, $z_{i}^{T}$) for an individual *i* is given as

$$\begin{array}{l} L\left(y_{i},m_{i},\delta_{i},x_{1i},\ldots ,x_{\left(k-1\right)i},z_{i}\right)\\ ={\left\{\text{Pr}\left(y_{i}|m_{i},x_{1i},\ldots ,x_{\left(k-1\right)i},z_{i}\right)f\left(m_{i}|x_{1i},\ldots ,x_{\left(k-1\right)i},z_{i}\right)\right\}}^{\delta_{i}}{\left\{\int_{m_{i}}^{\infty }\text{Pr}\left(y_{i}|t,x_{1i},\ldots ,x_{\left(k-1\right)i},z_{i}\right)dF\left(t|x_{1i},\ldots ,x_{\left(k-1\right)i},z_{i}\right)\right\}}^{1-\delta_{i}}\\ ={\left\{\frac{\text{exp}\left(-{\left(y_{i}-\left(b_{0}+bm_{i}+\sum_{j}{\tilde{c}}_{j}x_{ji}+\gamma^{T}z_{i}\right)\right)}^{2}/2\sigma^{2}\right)}{\sqrt{2\pi \sigma^{2}}}f\left(m_{i}|x_{1i},\ldots ,x_{\left(k-1\right)i},z_{i}\right)\right\}}^{\delta_{i}}{\left\{\int_{m_{i}}^{\infty }\frac{\text{exp}\left(-{\left(y_{i}-\left(b_{0}+bt+\sum_{j}{\tilde{c}}_{j}x_{ji}+\gamma^{T}z_{i}\right)\right)}^{2}/2\sigma^{2}\right)}{\sqrt{2\pi \sigma^{2}}}dF\left(t|x_{1i},\ldots ,x_{\left(k-1\right)i},z_{i}\right)\right\}}^{1-\delta_{i}}. \end{array}$$

We use the two-stage approach proposed in Wang et al. [18] to assess the coefficients for different paths *a*_*j*_, ${\tilde{c}}_{j}$, *j* = 1, …, *k* − 1, and *b*. In particular, in the first stage, based on the semiparametric AFT model [11,39,40], we use a weighted least square estimator to estimate coefficients *a*_*j*_, with the closed form as

$$\hat{\theta }={\left\{\sum_{i=1}^{n}\frac{\delta_{i}A_{i}A_{i}^{T}}{\hat{G}(m_{i})}\right\}}^{-1}\sum_{i=1}^{n}\frac{\delta_{i}A_{i}log(m_{i})}{\hat{G}(m_{i})},$$

where $\hat{G}(∙)$ is the Kaplan-Meier estimator for the censoring survival function which accounts for the right-censoring. Based on $\hat{\theta }={\left({\hat{a}}_{0},{\hat{a}}_{1},\ldots ,{\hat{a}}_{k-1},{\hat{\tilde{\gamma }}}^{T}\right)}^{T}$ and the AFT model error distribution ${\hat{\eta }}_{\hat{\theta }}(∙)$, which is estimated from the censored residues by Kaplan-Meier estimator, in the second stage, we assess the coefficients for paths *b*, and ${\tilde{c}}_{j}$, *j* = 1, …, *k* − 1, with the use of a log-pseudo-likelihood function as below, given a sample of *n* individuals:

$$PL\left(\varphi \right)=\frac{1}{n}\sum_{i=1}^{n}\left[\delta_{i}\;\text{log}\left\{{Pr}_{\varphi }\left(y_{i}|m_{i},A_{i}\right)\right\}+\left(1-\delta_{i}\right)log\int_{m_{i}-A_{i}^{T}\hat{\theta }}^{\tau }{Pr}_{\varphi }\left(y_{i}|t+A_{i}^{T}\hat{\theta },A_{i}\right)d{\hat{\eta }}_{\hat{\theta }}\left(t\right)\right],$$

where $\varphi ={\left(b_{0},b,{\tilde{c}}_{1},\ldots ,{{\tilde{c}}_{k-1},\gamma }^{T}\right)}^{T}$ is the set of parameters to be estimated and *τ* is the largest observed event time on a residual scale. The conditional probabilities Pr_*φ*_(*y*_*i*_|*m*_*i*_, *A*_*i*_) and ${Pr}_{\varphi }(y_{i}|t+A_{i}^{T}\hat{\theta },A_{i})$ can be formulated using the AFT model and the linear regression model. The estimators $\hat{\varphi }={\left({\hat{b}}_{0},\hat{b},{\hat{\tilde{c}}}_{1},\ldots ,{\hat{\tilde{c}}}_{k-1},{\hat{\gamma }}^{T}\right)}^{T}$ are assessed by maximizing the above log-pseudo-likelihood with the use of the minimization algorithms such as Nelder and Mead approach [41]. See Wang et al. [18] for details on parameter estimation.

We propose to assess the overall *IE*, *DE* and *TE* using an approach in which the *IE*s, *DE*s and *TE*s are calculated based on different categories of the exposure and combined using the corresponding frequencies of different categories of the exposure. The measures for *IE*, *DE*, and *TE* of each category of the exposure are derived following the counterfactual framework, which has been widely applied in mediation analysis, especially for scenarios involving nonlinearities and interactions [10,42–49]. Briefly, we denote $Y_{d_{j}}$ and $T_{d_{j}}$ respectively to be the values of the outcome *Y* and mediator *T* that would have been observed if the exposure *X* had been set to *d*_*j*_; and denote $Y_{d_{j}t}$ to be the value of *Y* that would have been observed if *T* and *X* had been set to *t* and *d*_*j*_ respectively [18,44,45]. Based on the counterfactual framework, conditional on the covariates *Z*, the natural *IE* is defined as $E\left(Y_{d_{j}T_{d_{j}}}|Z\right)-E\left(Y_{d_{j}T_{d_{0}}}|Z\right)$, which compares the effects of the mediator *T* at values of $T_{d_{j}}$ and $T_{d_{0}}$ on the outcome *Y* when the exposure *X* is set to value of *d*_*j*_; while the natural *DE* is defined as $E\left(Y_{d_{j}T_{d_{0}}}|Z\right)-E\left(Y_{d_{0}T_{d_{0}}}|Z\right)$, which compares the effects of the exposure *X* on *Y* by setting the mediator *T* to the value it would have been observed if *X* had been set to be *d*_0_ (reference category). Here, the assumptions on the absence of unmeasured confounders and consistency are required, which have been extensively discussed in the literature [8,12,13,18,43–48]. The detailed derivations for calculating *IE* and *DE* and associated assumptions are shown in the online S1 Appendix.

For each of the binary dummy-coded exposure variables *X*_*j*_, *j* = 1, …, *k* − 1, we evaluate the *IE* in the mediation model and denote it as *IE*_*j*_*versus*_0_ (indirect effect of category *X* = *d*_*j*_ versus the reference category *X* = *d*_0_), given *x*_*j*_ = 0 as the reference group:

$$IE_{j\_versus}{}_{\_0}=\begin{array}{c} \sum {}_{m}E\left(Y|m+a_{0}+a_{j}+\tilde{\gamma }z,x_{j}=1,z\right)\eta_{\theta }\left(m|x_{j}=1,z\right)-\\ \sum {}_{m}E\left(Y|m+a_{0}+\tilde{\gamma }z,x_{j}=1,z\right)\eta_{\theta }\left(m|x_{j}=0,z\right) \end{array}.$$

The overall indirect effect of the exposure *X* on the outcome *Y* mediated through the mediator *T* needs to account for the frequency of each possible categories *d*_*j*_ of *X*. Therefore, we define overall *IE* as:

$$IE=f_{1}{IE}_{1\_versus\_0}+f_{2}{IE}_{2\_versus\_0}+\cdots +f_{k-1}{IE}_{(k-1)\_versus\_0}=\sum_{j=1}^{k-1}f_{j}{IE}_{j\_versus\_0},$$ (1)

where *f*_*j*_, *j* = 1, …, *k* − 1, is the frequency of category *d*_*j*_ of the exposure. For genetic studies, *f*_*j*_ is the genotypic frequencies of possible genotypes of SNP *X* and can be obtained from external sources that represent general population such as the 1000 genome project data [42]; or one could estimate the genotypic frequencies from the current data.

If the models in the above equations are correctly specified, we can estimate the overall measures of *IE* based on the estimated AFT model error distribution for the mediator ${\hat{\eta }}_{\hat{\theta }}(\cdot )$ and the estimated coefficients $\hat{\theta }={\left({\hat{a}}_{0},{\hat{a}}_{1},\ldots ,{\hat{a}}_{k-1},{\hat{\tilde{\gamma }}}^{T}\right)}^{T}$ and $\hat{\varphi }={\left({\hat{b}}_{0},\hat{b},{\hat{\tilde{c}}}_{1},\ldots ,{\hat{\tilde{c}}}_{k-1},{\hat{\gamma }}^{T}\right)}^{T}$. On the basis of the counterfactual framework, the natural *IE*_*j*_*versus*_0_ for the dummy-coded exposure *X*_*j*_, as defined above, measures the expected change in outcome *Y* due to the effects of the mediator *T* at values of $T_{x_{j}=1}$ versus $T_{x_{j}=0}$ when *X*_*j*_ is set to 1, conditional on the covariates [18,50]. In turn, the overall *IE*, calculated accounting for the frequency of different categories of the exposure, measures the average expected change in *Y* from the effects of the mediator *T* responding to change/transition in the categorical exposure *X* from any group to the reference group [50].

We can similarly define direct effects, *DE*_*j*_*versus*_0_:

$$DE_{j_{-}versus_{-}0}=\begin{array}{c} \sum_{m}E\left(Y|m+a_{0}+\tilde{\gamma }z,x_{j}=1,z\right)\eta_{\theta }\left(m|x_{j}=0,z\right)-\\ \sum_{m}E\left(Y|m+a_{0}+\tilde{\gamma }z,x_{j}=0,z\right)\eta_{\theta }\left(m|x_{j}=0,z\right) \end{array}.$$

Similarly, the overall *DE* is then defined:

$$DE=\sum_{j=1}^{k-1}f_{j}{DE}_{j\_versus\_0}.$$ (2)

The overall *TE* is the summation of the *IE* and *DE*, calculated as *$TE=IE+DE=\sum_{j=1}^{k-1}f_{j}{IE}_{j\_versus\_0}+\sum_{j=1}^{k-1}f_{j}{DE}_{j\_versus\_0}$*. The proportion of total effect of *X* on *Y* mediated by the mediator *T* (*PM*) can be estimated as *PM* = *IE/TE*, which is commonly reported when there is a significant *IE* [51].

### *IE* for a mediation model with a categorical exposure and a binary outcome in a case-control study

We further extended the framework in the above section to accommodate a categorical exposure, a censored mediator and a binary outcome (e.g. presence or absence of a phenotype in case-control studies). The relationships among the variables can be specified using the logistic regression model and AFT model as follows:

$$\text{Logit}\{\text{Pr}(y_{i}=1|t_{i},x_{1i},\ldots ,x_{\left(k-1\right)i})\}=b_{0}+bt_{i}+\sum_{j=1}^{k-1}{\tilde{c}}_{j}x_{ji}+\gamma^{T}z_{i},$$

and

$$log\left(t_{i}\right)=a_{0}+\sum_{j=1}^{k-1}a_{j}x_{ji}+{\tilde{\gamma }}^{T}z_{i}+\varepsilon_{ti}=A_{i}^{T}\theta +\varepsilon_{ti}.$$

All coefficients are defined similarly as for the scenario with a continuous outcome. To account for the binary nature of the outcome variable in a case-control study where cases may be oversampled, in stage one, in addition to the weight to account for the right-censoring, we consider one additional sampling weight, i.e. *w*_*i*_, in the weighted least square estimator to estimate coefficients *a*_*j*_, *j* = 1, …, *k* − 1, as below:

$$\hat{\theta }={\left\{\sum_{i=1}^{n}\frac{\delta_{i}w_{i}A_{i}A_{i}^{T}}{\hat{G}(m_{i})}\right\}}^{-1}\sum_{i=1}^{n}\frac{\delta_{i}w_{i}A_{i}log(m_{i})}{\hat{G}(m_{i})}.$$

The log-pseudo-likelihood function can be similarly defined by including the sampling weight:

$$PL\left(\varphi \right)=\frac{1}{n}\sum_{i=1}^{n}w_{i}\left[\delta_{i}\;\text{log}\left\{{Pr}_{\varphi }\left(y_{i}|m_{i},A_{i}\right)\right\}+\left(1-\delta_{i}\right)log\int_{m_{i}-A_{i}^{T}\hat{\theta }}^{\tau }{Pr}_{\varphi }\left(y_{i}|t+A_{i}^{T}\hat{\theta },A_{i}\right)d{\hat{\eta }}_{\hat{\theta }}\left(t\right)\right],$$

where the coefficients $\varphi ={\left(b_{0},b,{\tilde{c}}_{1},\ldots ,{{\tilde{c}}_{k-1},\gamma }^{T}\right)}^{T}$ can be evaluated to provide the coefficients for paths *b* and ${\tilde{c}}_{j}$, *j* = 1, …, *k* − 1. Specifically, the coefficients ${\tilde{c}}_{j}$, *j* = 1, …, *k* − 1, correspond to the paths from *k*-1 dummy variables created for the exposure, *X*_1_, *X*_2_, …, *X*_*k*−1_, to the outcome *Y*, and *b* corresponds to the path from the mediator *T* to the outcome *Y*. The sampling weight *w*_*i*_ is included to account for the sampling mechanism of case-control study design. Such weighting strategy of using inverse disease prevalence is well-established [18,52–58].

To assess the overall *IE* and *DE*, we first estimate the *IE* and *DE* for each of the dummy-coded exposure variables *X*_*j*_, *j* = 1, …, *k* − 1, as described above, *IE*_*j*_*versus*_0_ and *DE*_*j*_*versus*_0_:

$$IE_{j_{-}versus_{-}0}=\begin{array}{c} \sum_{m}\text{Pr}\left(Y=1|m+a_{0}+a_{j}+\tilde{\gamma }z,x_{j}=1,z\right)\eta_{\theta }\left(m|x_{j}=1,z\right)-\\ \sum_{m}\text{Pr}\left(Y=1|m+a_{0}+\tilde{\gamma }z,x_{j}=1,z\right)\eta_{\theta }\left(m|x_{j}=0,z\right) \end{array},$$

and

$$DE_{j_{-}versus_{-}0}=\begin{array}{c} \sum_{m}\text{Pr}\left(Y=1|m+a_{0}+\tilde{\gamma }z,x_{j}=1,z\right)\eta_{\theta }\left(m|x_{j}=0,z\right)-\\ \sum_{m}\text{Pr}\left(Y=1|m+a_{0}+\tilde{\gamma }z,x_{j}=0,z\right)\eta_{\theta }\left(m|x_{j}=0,z\right) \end{array},$$

Based on the estimated AFT model error distribution for the mediator ${\hat{\eta }}_{\hat{\theta }}(\cdot )$ and the estimated coefficients $\hat{\theta }={\left({\hat{a}}_{0},{\hat{a}}_{1},\ldots ,{\hat{a}}_{k-1},{\hat{\tilde{\gamma }}}^{T}\right)}^{T}$ and $\hat{\varphi }={\left({\hat{b}}_{0},\hat{b},{\hat{\tilde{c}}}_{1},\ldots ,{\hat{\tilde{c}}}_{k-1},{\hat{\gamma }}^{T}\right)}^{T}$, the overall *IE* and *DE* of the exposure *X* for the mediation model are calculated using Eqs (1) and (2).

The study and data use were approved by US National Institute of Health and The University of Texas MD Anderson Cancer Center through Material Transfer Agreement (MTA ID: 00016197). The data of the motivating study was downloaded from dbGaP (phs000209.v13.p3) [59] for the Multi-Ethnic Study of Atherosclerosis (MESA) cohort study. The MESA study was approved by the Institutional Review Board at each site of the study and informed consent was obtained from each participant [60].

## Simulation

### Simulation approach

To examine the performance of the proposed overall measures of *IE*, *DE*, and *TE*, we conducted simulations for a mediation model with a categorical exposure, where the mediator is subject to right censoring.

#### Binary outcome

To mimic the motivation study, we first considered a case-control study in the simulations, with a binary outcome and an additive genetic variant (SNP) as the exposure. We used the robust estimating approach by Wang et al. [18] to estimate the parameters under the mediation model. For each individual *i*, the genotype of the genetic variant *x*_*i*_ (exposure) was generated with the use of the genotypes’ frequencies assuming the genetic variant is in Hardy-Weinberg proportion. We assumed a genetic variant with a minor allele frequency (MAF) of 0.1, 0.3 and 0.5. The genotypic frequencies could be calculated accordingly. For example, when the MAF was 0.3, the genotypic frequencies were 0.49, 0.42, and 0.09 for the three genotypes *rr*, *rR*, and *RR*, respectively. Given the exposure *x*_*i*_, the censored mediator *t*_*i*_ was generated using an AFT model log(*t*_*i*_) = *a*_0_ + *ax*_*i*_ + *ε*_*t*_, where *ε*_*t*_ ~ Normal(0, 1) and the coefficients were set as *a*_0_ = 6 and *a*_0_ = 0 or 0.4. The right-censoring time *c*_*i*_ was generated independently from the uniform distributions using different intervals to create different censoring percentages, ~20% and ~40%. The observed censored variable *m*_*i*_ and indicator *δ*_*i*_ for each individual were then obtained as *m*_*i*_ = min(*t*_*i*_, *c*_*i*_) and *δ*_*i*_ = *I*(*t*_*i*_ ≤ *c*_*i*_). Conditioned on the values of *x*_*i*_ and *t*_*i*_, the outcome *y*_*i*_ was generated using the logistic regression model, where the coefficients were set to be *b* = 0 or 0.4, and $\tilde{c}=0.5$. The intercept coefficient *b*_0_ was set to various values to reflect different disease prevalence of ~10% and ~30%. In this way, we simulated a large amount of data on the population, from which we randomly sampled same numbers of cases and controls based on the outcome status. In particular, we randomly sampled 500 cases and controls for the scenarios where MAF = 0.3 and 0.5; while sampled 2000 cases and controls for the scenarios where MAF = 0.1 to ensure the sufficient sample size in *RR* genotype category, thereby, producing stable estimations of the regression coefficients.

Note that in the simulations, we employed *X* = (0, 1, 2) corresponding to the genotypes (*rr*, *rR*, *RR*). In this situation, the regression coefficients, *a* and $\tilde{c}$, are interpreted as the per-allele effect, which corresponds to the effect of each copy of the deleterious allele *R*. When analyzing the mediation model, as described in the Methods section, we created two dummy variables, *X*_1_ = (0, 1, 0) and *X*_2_ = (0, 0, 1) using the genotype *rr* as the reference group. Given such a coding, it is straightforward to derive that *a*_1_ = *a*, *a*_2_ = 2*a*, ${\tilde{c}}_{1}=c$, and ${\tilde{c}}_{2}=2c$. That is, *a*_1_ = 0 or 0.4; *a*_2_ = 0 or 0.8; ${\tilde{c}}_{1}=0.5$; and ${\tilde{c}}_{2}=1$. We report the estimates for *a*_1_, *a*_2_, ${\tilde{c}}_{1}$, and ${\tilde{c}}_{2}$ for the simulation studies.

For each of the MAF values (i.e., 0.1, 0.3, 0.5), when either coefficient *a* = 0 or *b* = 0, we considered twelve scenarios for which there is no *IE* through the mediator, with respect to different censoring percentages (~20 or 40%) and different disease prevalence (~10 or 30%). When both *a* and *b* are non-zero (i.e., *a* = *b* = 0.4), we considered four scenarios for which there is an *IE* through the mediator with respect to different censoring percentages and disease prevalence. For different scenarios, the theoretical true values of *IE*s and *PM*s were calculated using the prespecified parameters and prespecified normal distribution for the conditional probability of the mediator. In our estimating procedure to calculate the empirical *IE*s and *PM*s, the nonparametric Kaplan-Meier estimator of the censored residuals was used to assess the conditional probability of the mediator. To test the significance of the *IE*s and *PM*s, the bias-corrected and accelerated (BCa) bootstrap approach [61] was employed to determine the confidence intervals (CIs) for the *IE*s and *PM*s [17,18].

#### Continuous outcome

When investigating the performance of the proposed overall measures for a mediation model with a continuous outcome, a categorical exposure (genetic variant) and a censored mediator, we generated the exposure *X* and the mediator *T* similarly as described above. We still assumed different MAFs for the genetic variant (i.e., 0.1, 0.3, 0.5) and different censoring percentages for the mediator (i.e., ~20%, ~40%). For each individual *i*, given the values of the exposure *x*_*i*_ and the mediator *t*_*i*_, the outcome *y*_*i*_ was generated using the linear regression model. The coefficients were set to be *b*_0_ = 1, *b* = 0 or 0.4, and $\tilde{c}=0.5$. We randomly generated 1000 samples for the scenarios where MAF = 0.3 and 0.5; and 4000 samples for the scenarios where MAF = 0.1. For each of the MAF values (i.e., 0.1, 0.3, 0.5), there were six scenarios for which there is no *IE* through the mediator (i.e., *a* = 0 or *b* = 0); and two scenarios for which there is an *IE* through the mediator (i.e., *a* = *b* = 0.4), with respect to different censoring percentages of the mediator.

### Simulation results

#### Binary outcome

Tables 1 and 2 report the simulation results for all the scenarios assuming a binary outcome in a case-control study and an additive SNP with MAF = 0.3, including the scenarios for which there is no *IE* through the mediator (top panel) and those for which there is an *IE* through the mediator (bottom panel). In Table 1, we report the means and standard errors of the estimated coefficients for the different paths, *a*_0_, *a*_1_, *a*_2_, *b*_0_, *b*, ${\tilde{c}}_{1}$, and ${\tilde{c}}_{2}$. As expected, the robust approach provided accurate estimations for all coefficients through different scenarios. As an example of no *IE*, scenario 3, in which the prespecified values were *a*_*0*_ = 6, *a* = 0 (i.e., *a*_1_ = 0, *a*_2_ = 0), *b*_*0*_ = -5 (i.e., disease prevalence = ~10%), *b* = 0.4, and $\tilde{c}=0.5$ (i.e., ${\tilde{c}}_{1}=0.5$, ${\tilde{c}}_{2}=1$), the estimated values were *a*_*0*_ = 5.9995, *a*_1_ = 0.0019, *a*_2_ = 0.0080, *b*_0_ = -5.0064, *b* = 0.3984, ${\tilde{c}}_{1}=0.5057$ and ${\tilde{c}}_{2}=1.0055$, respectively, which were close to the prespecified parameter values in the simulation model. Similarly, under the scenarios where there is an *IE* through the mediator, all parameters were accurately estimated. For example, for scenario 15, in which the prespecified values were *a*_0_ = 6, *a* = 0.4 (i.e., *a*_1_ = 0.4, *a*_2_ = 0.8), *b*_*0*_ = -3.7 (i.e., disease prevalence = ~30%), *b* = 0.4, and $\tilde{c}=0.5$ (i.e., ${\tilde{c}}_{1}=0.5$, ${\tilde{c}}_{2}=1$), the estimated values were *a*_0_ = 6.0043, *a*_1_ = 0.3985, *a*_2_ = 0.7809, *b*_0_ = -3.6902, *b* = 0.3975, ${\tilde{c}}_{1}=0.5004$ and ${\tilde{c}}_{2}=1.0074$, respectively, which were close to the prespecified parameter values in the simulation model.

**Table 1:** Binary outcome: Means and standard errors (se) of estimated coefficients for different paths, *a*_0_, *a*_1_, *a*_2_, *b*_0_, *b*, ${\tilde{c}}_{1}$ and ${\tilde{c}}_{2}$, given the minor allele frequency (MAF) = 0.3.

Scenario	*a*	*b*_*0*_	*b*	Theoretical *IE*	Theoretical *PM*	*CP*	prev	Estimated Parameters						
								*a*_*0*_ (*se*)	*a*_*1*_ (*se*)	*a*_*2*_ (*se*)	*b*_*0*_ (*se*)	*b* (*se*)	${\tilde{c}}_{1}$ (*se*)	${\tilde{c}}_{2}$ (*se*)
**Without *IE***														
1	0	-2.5	0	0.000	0.000	21%	10%	6.0008 (0.066)	-0.0028 (0.099)	-0.0048 (0.162)	-2.5292 (0.441)	0.0042 (0.072)	0.5023 (0.135)	1.0173 (0.230)
2	0	-2.5	0	0.000	0.000	40%	10%	5.9933 (0.080)	0.0042 (0.113)	-0.0059 (0.197)	-2.4970 (0.499)	-0.0001 (0.081)	0.4879 (0.137)	1.0057 (0.215)
3	0	-5	0.4	0.000	0.000	21%	10%	5.9995 (0.064)	0.0019 (0.094)	0.0080 (0.159)	-5.0064 (0.479)	0.3984 (0.075)	0.5057 (0.144)	1.0055 (0.221)
4	0	-5	0.4	0.000	0.000	36%	10%	6.0047 (0.072)	-0.0057 (0.115)	-0.0077 (0.180)	-5.0142 (0.502)	0.3982 (0.079)	0.5049 (0.149)	1.0229 (0.211)
5	0.4	-2.5	0	0.000	0.000	20%	10%	6.0008 (0.065)	0.3972 (0.098)	0.7943 (0.163)	-2.5287 (0.440)	0.0041 (0.072)	0.5007 (0.137)	1.0144 (0.237)
6	0.4	-2.5	0	0.000	0.000	42%	10%	5.9933 (0.080)	0.4039 (0.116)	0.7873 (0.205)	-2.4913 (0.511)	-0.0010 (0.083)	0.4885 (0.138)	1.0063 (0.223)
7	0	-1.2	0	0.000	0.000	21%	29%	6.0009 (0.059)	0.0002 (0.086)	-0.0053 (0.140)	-1.2027 (0.404)	0.0000 (0.068)	0.5068 (0.131)	1.0163 (0.226)
8	0	-1.2	0	0.000	0.000	40%	29%	5.9959 (0.062)	-0.0005 (0.091)	0.0004 (0.164)	-1.2323 (0.493)	0.0047 (0.081)	0.5069 (0.133)	1.0081 (0.230)
9	0	-3.7	0.4	0.000	0.000	21%	28%	5.9981 (0.057)	-0.0014 (0.085)	-0.0008 (0.136)	-3.7022 (0.444)	0.3988 (0.071)	0.5071 (0.134)	1.0216 (0.240)
10	0	-3.7	0.4	0.000	0.000	40%	28%	6.0060 (0.064)	-0.0074 (0.092)	-0.0155 (0.155)	-3.7249 (0.509)	0.4017 (0.082)	0.5001 (0.138)	1.0281 (0.247)
11	0.4	-1.2	0	0.000	0.000	20%	29%	6.0007 (0.058)	0.4011 (0.084)	0.7954 (0.143)	-1.1998 (0.403)	-0.0005 (0.067)	0.5069 (0.136)	1.0165 (0.236)
12	0.4	-1.2	0	0.000	0.000	39%	29%	5.9973 (0.061)	0.3978 (0.088)	0.8002 (0.167)	-1.2282 (0.491)	0.0040 (0.081)	0.5052 (0.136)	1.0047 (0.239)
**With *IE***														
13	0.4	-5.2	0.4	0.021	0.311	20%	9%	5.9987 (0.065)	0.3988 (0.096)	0.7987 (0.155)	-5.1956 (0.488)	0.3978 (0.076)	0.4897 (0.148)	1.0042 (0.228)
14	0.4	-5.2	0.4	0.021	0.311	39%	9%	5.9924 (0.071)	0.4073 (0.110)	0.8013 (0.187)	-5.2247 (0.616)	0.4009 (0.095)	0.4914 (0.148)	1.0196 (0.243)
15	0.4	-3.7	0.4	0.043	0.268	20%	30%	6.0043 (0.052)	0.3985 (0.076)	0.7809 (0.131)	-3.6902 (0.455)	0.3975 (0.074)	0.5004 (0.150)	1.0074 (0.255)
16	0.4	-3.7	0.4	0.043	0.268	39%	30%	6.0006 (0.062)	0.4028 (0.096)	0.7983 (0.155)	-3.7448 (0.521)	0.4059 (0.084)	0.4892 (0.145)	1.0160 (0.256)

The simulation was based on 500 replicates, each with 500 cases and 500 controls. Different scenarios were considered based on different values of *a*, *b*_*0*_, *b*, censoring percentage (*CP*) and disease prevalence (prev), with *a*_*0*_ = 6 and $\tilde{c}=0.5$.

Abbreviations: IE, indirect effect; PM: Proportions of the total effect mediated.

**Table 2:** Binary outcome: Means and standard errors (se) of indirect effects (*IEs*) and proportions of the total effect mediated (*PMs*); and coverage probabilities (cov) of the 95% confidence intervals for the estimations of *IE* and *PM*, given the minor allele frequency (MAF) = 0.3.

Scenario	*a*	*b*_*0*_	*b*	Theoretical *IE*	Theoretical *PM*	*CP*	prev	Estimated Mediating Effects			
								*IE* (*se*)	95% cov	*PM* (*se*)	95% cov
**Without *IE***											
1	0	-2.5	0	0.000	0.000	21%	10%	-0.0001 (0.001)	0.990	-	-
2	0	-2.5	0	0.000	0.000	40%	10%	-0.0002 (0.001)	0.998	-	-
3	0	-5	0.4	0.000	0.000	21%	10%	0.0000 (0.004)	0.938	-	-
4	0	-5	0.4	0.000	0.000	36%	10%	-0.0005 (0.005)	0.930	-	-
5	0.4	-2.5	0	0.000	0.000	20%	10%	0.0000 (0.004)	0.944	-	-
6	0.4	-2.5	0	0.000	0.000	42%	10%	-0.0003 (0.005)	0.927	-	-
7	0	-1.2	0	0.000	0.000	21%	29%	-0.0001 (0.001)	0.998	-	-
8	0	-1.2	0	0.000	0.000	40%	29%	-0.0001 (0.002)	0.998	-	-
9	0	-3.7	0.4	0.000	0.000	21%	28%	-0.0002 (0.007)	0.940	-	-
10	0	-3.7	0.4	0.000	0.000	40%	28%	-0.0010 (0.008)	0.952	-	-
11	0.4	-1.2	0	0.000	0.000	20%	29%	-0.0002 (0.007)	0.938	-	-
12	0.4	-1.2	0	0.000	0.000	39%	29%	0.0003 (0.009)	0.928	-	-
**With *IE***											
13	0.4	-5.2	0.4	0.021	0.311	20%	9%	0.0217 (0.005)	0.954	0.3122 (0.080)	0.954
14	0.4	-5.2	0.4	0.021	0.311	39%	9%	0.0219 (0.006)	0.948	0.3134 (0.087)	0.958
15	0.4	-3.7	0.4	0.043	0.268	20%	30%	0.0418 (0.009)	0.962	0.2689 (0.079)	0.938
16	0.4	-3.7	0.4	0.043	0.268	39%	30%	0.0430 (0.011)	0.948	0.2767 (0.083)	0.946

The simulation was based on 500 replicates, each with 500 cases and 500 controls. Different scenarios were considered based on different values of *a*, *b*_*0*_, *b*, censoring percentage (*CP*) and disease prevalence (prev), with *a*_*0*_ = 6 and $\tilde{c}=0.5$.

Table 2 report the means and standard errors of the estimated *IE*s and *PM*s (for the scenarios with significant *IE*s) for MAF = 0.3, obtained using the overall measures proposed in the study, and the coverage probabilities of the 95% CIs of the *IE*s and *PM*s. When estimating the *IE*s and *PM*s, our overall measures provided accurate estimations for all scenarios. For example, for scenario 16, when the theoretical *IE* and *PM* were respectively 0.043 and 0.268, the estimated values obtained using our approach were 0.0430 and 0.2767, which were close to the theoretical values. The 95% coverage probabilities for the *IE* and *PM*, based on the proposed approach, were close to the nominal value of 0.95. The proposed measures were practically not impacted by different disease prevalence values (~10 or 30%) and censoring percentages (~20 or 40%).

For the other scenarios where the MAFs of the genetic variant (exposure) were 0.1 and 0.5, the simulation results are reported in the online S1 and S2 Tables, respectively. Similar results were observed. The proposed approach provided accurate estimations for all coefficients of different paths, as well as *IE*s and *PM*s, through various scenarios. It is worth to note that a relatively small MAF for the genetic variant (e.g., 10%) would affect the parameter estimations for the paths *a*_2_, and ${\tilde{c}}_{2}$, which were particularly pronounced when sample size was smaller (e.g., 500 cases and controls; data not shown). This is not surprising to observe because in this situation, the expected frequency of the genotype *RR* is only 1%, resulting in a very small number of samples in this category. Larger sample sizes can help to ensure the accurate estimations of these parameters. Therefore, when MAF = 0.1, we increased the sample size to 2000 cases and 2000 controls (or 4000 samples for the continuous outcome) in the simulation studies.

#### Continuous outcome

Tables 3 and 4 report the simulation results for all the scenarios assuming a continuous outcome and an additive SNP with MAF = 0.3. Similar to the scenarios with a binary outcome, the proposed approach provided accurate estimations for all coefficients, *IE*s and *PM*s, for mediation models with a continuous outcome, regardless of different values of *a*, *b*, and censoring percentage. For example, for scenario 8, where the prespecified values for simulation were *a*_*0*_ = 6, *a* = 0.4 (i.e., *a*_1_ = 0.4, *a*_2_ = 0.8), *b*_*0*_ = 1, *b* = 0.4, and $\tilde{c}=0.5$ (i.e., ${\tilde{c}}_{1}=0.5$, ${\tilde{c}}_{2}=1$), the estimated values were *a*_0_ = 5.9954, *a*_1_ = 0.4004, *a*_2_ = 0.7979, *b*_*0*_ = 1.0084, *b* = 0.3990, ${\tilde{c}}_{1}=0.4987$, and ${\tilde{c}}_{2}=0.9944$, respectively, which were close to the prespecified parameter values (Table 3). Meanwhile, the estimated overall *IE* and *PM* were 0.1877 and 0.2433, respectively, which were close to the theoretical values of 0.188 and 0.242 (Table 4). The 95% coverage probabilities for the *IE* and *PM* were 0.948 and 0.944 and both were close to the nominal value of 0.95. The simulation results for scenarios where MAF = 0.1 and 0.5 are reported in the online S3 and S4 Tables, respectively. As in the binary outcome scenarios, we increased the sample size to 4000 when MAF = 0.1 for the genetic variant.

**Table 3:** Continuous outcome: Means and standard errors (se) of estimated coefficients for different paths, *a*_*0*_, *a*_*1*_, *a*_*2*_, *b*_*0*_, *b*, ${\tilde{c}}_{1}$ and ${\tilde{c}}_{2}$, given the minor allele frequency (MAF) = 0.3.

Scenario	*a*	*b*	Theoretical *IE*	Theoretical *PM*	*CP*	Estimated Parameters						
						*a*_*0*_ (*se*)	*a*_*1*_ (*se*)	*a*_*2*_ (*se*)	*b*_*0*_ (*se*)	*b* (*se*)	${\tilde{c}}_{1}$ (*se*)	${\tilde{c}}_{2}$ (*se*)
**Without *IE***												
1	0	0	0.000	0.000	21%	5.9990 (0.052)	0.0022 (0.075)	-0.0080 (0.125)	0.9899 (0.210)	0.0011 (0.034)	0.5046 (0.066)	1.0030 (0.114)
2	0	0	0.000	0.000	40%	5.9980 (0.058)	0.0081 (0.087)	-0.0039 (0.159)	1.0108 (0.232)	-0.0019 (0.038)	0.4999 (0.065)	0.9990 (0.115)
3	0	0.4	0.000	0.000	21%	5.9989 (0.051)	0.0007 (0.080)	-0.0012 (0.128)	1.0058 (0.207)	0.3995 (0.034)	0.4938 (0.070)	0.9938 (0.115)
4	0	0.4	0.000	0.000	36%	5.9955 (0.058)	0.0050 (0.087)	0.0075 (0.144)	0.9978 (0.222)	0.4004 (0.036)	0.5016 (0.069)	1.0041 (0.116)
5	0.4	0	0.000	0.000	20%	6.0032 (0.050)	0.3973 (0.073)	0.7999 (0.133)	0.9916 (0.213)	0.0010 (0.035)	0.5053 (0.071)	0.9999 (0.117)
6	0.4	0	0.000	0.000	42%	5.9984 (0.058)	0.3996 (0.094)	0.7856 (0.166)	0.9976 (0.238)	0.0007 (0.039)	0.4989 (0.068)	0.9847 (0.118)
**With *IE***												
7	0.4	0.4	0.188	0.242	20%	6.0025 (0.052)	0.3964 (0.078)	0.7907 (0.133)	1.0150 (0.214)	0.3974 (0.035)	0.5033 (0.070)	1.0047 (0.120)
8	0.4	0.4	0.188	0.242	39%	5.9954 (0.053)	0.4004 (0.083)	0.7979 (0.154)	1.0084 (0.237)	0.3990 (0.039)	0.4987 (0.072)	0.9944 (0.124)

The simulation was based on 500 replicates, each with 1000 samples. Different scenarios were considered based on different values of *a*, *b*, and censoring percentage (*CP*), with *a*_*0*_ = 6, *b*_*0*_ = 1 and $\tilde{c}=0.5$.

Abbreviations: IE, indirect effect; PM: Proportions of the total effect mediated.

**Table 4:** Continuous outcome: Means and standard errors (se) of indirect effects (*IEs*) and proportions of the total effect mediated (*PMs*); and coverage probabilities (cov) of the 95% confidence intervals for the estimations of *IE* and *PM*, given the minor allele frequency (MAF) = 0.3.

Scenario	*a*	*b*	Theoretical *IE*	Theoretical *PM*	*CP*	Estimated Mediating Effects			
						*IE* (*se*)	95% cov	*PM* (*se*)	95% cov
**Without *IE***									
1	0	0	0.000	0.000	21%	-0.0001 (0.002)	0.998	-	-
2	0	0	0.000	0.000	40%	-0.0001 (0.003)	0.996	-	-
3	0	0.4	0.000	0.000	21%	0.0000 (0.031)	0.922	-	-
4	0	0.4	0.000	0.000	36%	0.0021 (0.032)	0.954	-	-
5	0.4	0	0.000	0.000	20%	0.0003 (0.016)	0.938	-	-
6	0.4	0	0.000	0.000	42%	0.0005 (0.019)	0.946	-	-
**With *IE***									
7	0.4	0.4	0.188	0.242	20%	0.1850 (0.034)	0.922	0.2390 (0.044)	0.934
8	0.4	0.4	0.188	0.242	39%	0.1877 (0.035)	0.948	0.2433 (0.045)	0.944

The simulation was based on 500 replicates, each with 1000 samples. Different scenarios were considered based on different values of *a*, *b*, and censoring percentage (*CP*), with *a*_*0*_ = 6, *b*_*0*_ = 1 and $\tilde{c}=0.5$.

## Application to the motivation study

We applied the proposed overall measures for *IE*, *DE*, and *TE* for the mediation analysis to the data from a genetic case-control study of type 2 diabetes downloaded from dbGaP [59], relating to the Multi-Ethnic Study of Atherosclerosis (MESA) cohort study. The conceptual mediation model is shown in Fig 1, where the genetic variant is the exposure (*X*), the age at menopause is the mediator (*T*), and type 2 diabetes status is the outcome variable (*Y*).

There were 47,871 genetic variants from 2,956 women included in the mediation analysis. A woman’s age at menopause was censored if she had not gone through menopause, and the censoring percentage was ~14.5%. Assuming an additive genetic model for all the genetic variants, we conducted the association analyses of genetic variants with a woman’s age at menopause (path *a*) as well as with type 2 diabetes status (paths *b* and $\tilde{c}$). In particular, when assessing the association between a woman’s age at menopause and a genetic variant with type 2 diabetes (paths *b* and $\tilde{c}$), we used logistic regression model, where type 2 diabetes status was the dependent variable and the genetic variant and age at menopause were the predictors. We included age and ethnicity as covariates in the logistic regression model. When assessing the association between a genetic variant with a woman’s age at menopause (path *a*), we used the AFT model, where age at menopause was the dependent variable and the genetic variant was the predictor. Ethnicity was adjusted as a covariate in the AFT model. There were four categories for the ethnicity in the MESA data, including White, Caucasian; Black, African-American; Chinese American; and Hispanic. Ethnicity was considered as a categorical variable in the analysis where White, Caucasian was used as the reference category, resulting in three related coefficients in the AFT model (${\tilde{\gamma }}_{1}$, ${\tilde{\gamma }}_{2}$ and ${\tilde{\gamma }}_{3}$) and the logistic regression model (*γ*_1_, *γ*_2_ and *γ*_3_). Age was considered as a continuous variable, resulting in one coefficient (*γ*_4_) in the logistic regression model.

For the purpose of demonstration, we considered a threshold of 0.005 to identify top variants. Our approach identified three variants—rs12744291, rs2503182, and rs11771343—associated with both type 2 diabetes and age at menopause to be included in the mediation analysis. Specifically, the *p*-values were 1.27×10^−3^, 2.56×10^−3^, and 1.43×10^−3^ for rs12744291, rs2503182, and rs11771343, respectively, for their association with type 2 diabetes and 5.53×10^−4^, 4.01×10^−3^, and 4.12×10^−3^, respectively, for their association with age at onset of menopause. The top and middle panels of Table 5 list the estimations for all the coefficients for the AFT model and logistic regression model, respectively, for the three top genetic variants. Consider SNP rs12744291 as an example, in the AFT model where age at menopause was the dependent variable, the estimated coefficients were *a*_1_ = 0.8768 and *a*_2_ = 1.4933 for the SNP (path *a*); and ${\tilde{\gamma }}_{1}=0.6625$, ${\tilde{\gamma }}_{2}=-0.7286$ and ${\tilde{\gamma }}_{3}=-1.0543$ for ethnicity. In the logistic regression model where type 2 diabetes was the dependent variable, the estimated coefficients were *b* = -0.0090, ${\tilde{c}}_{1}=-0.1704$, ${\tilde{c}}_{2}=-0.8943$ for the age at menopause and SNP (paths *b* and $\tilde{c}$); and *γ*_1_ = 1.4437, *γ*_2_ = 1.4859, *γ*_3_ = 1.7071, and *γ*_4_ = 0.0305 for ethnicity and age.

**Table 5:** Estimations of the coefficients, as well as the overall total effects (*TEs*), direct effects (*DEs*), and indirect effects (*IEs*), along with 95% confidence intervals (CIs), for the single nucleotide polymorphisms (SNPs) associated with both type 2 diabetes and a woman’s age at menopause in the real data analysis*.

**AFT model: association of SNPs with a woman’s age at menopause**									
			**Age at menopause**	**SNP**		**Ethnicity**			**Age**
**CHR**	**SNP**	***a***_***0***_ **(CI)**	**-**	***a***_***1***_ **(CI)**	***a***_***2***_ **(CI)**	${\tilde{\gamma }}_{1}$ **(CI)**	${\tilde{\gamma }}_{2}$ **(CI)**	${\tilde{\gamma }}_{3}$ **(CI)**	**-**
1	rs12744291	48.2383 [47.77,48.68]	-	0.8768 [0.21,1.54]	1.4933 [0.49,2.37]	0.6625 [-0.15,1.38]	-0.7286 [-1.60,-0.02]	-1.0543 [-1.83,-0.17]	-
1	rs2503182	48.4052 [47.96,48.86]	-	0.5798 [-0.03,1.16]	1.5801 [0.34,2.97]	0.8090 [-0.06,1.61]	-0.8548 [-1.78,-0.04]	-0.9349 [-1.64,-0.18]	-
7	rs11771343	49.0283 [48.58,49.47]	-	-0.6597 [-1.36,-0.02]	-1.0868 [-1.95,0.02]	1.0655 [0.20,1.73]	-0.3479 [-1.52,0.54]	-0.9968 [-1.90,-0.30]	-
**Logistic regression model: association of SNPs and a woman’s age at menopause with type 2 diabetes**									
			**Age at menopause**	**SNP**		**Ethnicity**			**Age**
**CHR**	**SNP**	***b***_***0***_ **(CI)**	***b* (CI)**	${\tilde{c}}_{1}$ **(CI)**	${\tilde{c}}_{2}$ **(CI)**	***γ***_**1**_ **(CI)**	***γ***_**2**_ **(CI)**	***γ***_**3**_ **(CI)**	***γ***_**4**_ **(CI)**
1	rs12744291	-4.5419 [-6.22,-3.38]	-0.0090 [-0.03,0.01]	-0.1704 [-0.43,0.11]	-0.8943 [-1.54,-0.38]	1.4437 [0.96,1.84]	1.4859 [1.11,1.81]	1.7071 [1.34,2.12]	0.0305[0.02, 0.04]
1	rs2503182	-4.6732 [-6.24,-3.67]	-0.0078 [-0.03,0.01]	-0.2587 [-0.55,-0.01]	-0.9561 [-2.10,-0.39]	1.3750 [0.93,1.89]	1.4857 [1.11,1.85]	1.6363 [1.31,2.00]	0.0321 [0.02,0.05]
7	rs11771343	-5.1460 [-6.83,-3.55]	-0.0063 [-0.03,0.01]	0.4255 [0.07,0.73]	0.5624 [0.21,0.93]	1.2643 [0.83,1.75]	1.2940 [0.83,1.73]	1.6725 [1.32,2.04]	0.0324 [0.02,0.04]
***TE*, *DE*, and *IE***									
**CHR**	**SNP**	***TE***	**CI *of TE***	***DE***	**CI of *DE***	***IE***	**CI of I*E***		
1	rs12744291	-0.0275	[-0.0511,-0.0024]	-0.0267	[-0.0498,-0.0024]	-0.0007	[-0.0037,0.0004]		
1	rs2503182	-0.0309	[-0.0549,-0.0101]	-0.0305	[-0.0545,-0.0096]	-0.0004	[-0.0018,0.0008]		
7	rs11771343	0.0432	[0.0119,0.0639]	0.0427	[0.0117,0.0647]	0.0005	[-0.0010,0.0029]		

The 95% confidence intervals (CIs) were assessed using a bootstrap approach with 200 bootstraps.

* Proportions of total effects mediated by the mediator are not reported because the indirect effects are nonsignificant.

Abbreviation: CHR, chromosome.

The bottom panel of Table 5 reports the overall *IE*s, *DE*s, *TE*s, and 95% CIs obtained from the mediation analysis of the three genetic variants, a woman’s age at menopause, and type 2 diabetes. BCa bootstrapping was used to assess the CIs for *IE*s as in the simulation studies. The overall *IE*s for the three genetic variants, rs12744291, rs2503182, and rs11771343, were reported as -0.0007, -0.0004, and 0.0005, respectively; and the 95% CIs of *IE*s for all three genetic variants include zero. These results suggest no statistically significant mediating effect of the age at menopause on the association between the three variants and type 2 diabetes risk.

## Discussion

In this study, we proposed overall measures to calculate the *IE*, *DE*, and *TE* for a single censored mediator model involving a categorical exposure. Specifically, we defined the *IE*, *DE*, and *TE* for each of the categories for the exposure first and then assessed the overall *IE*, *DE*, and *TE* of the exposure accounting for the frequencies of different categories of the categorical exposure variable.

Compared with the traditional approach for a multi-categorical exposure, the proposed measure has several advantages. First, it provides an overall *IE*, *DE*, and *TE* of the mediation model from the exposure, instead of relative *IE*, *DE*, and *TE* as described in previous studies. Second, it avoids the multiple testing issue caused by recoding the multi-categorical exposure into multiple binary exposure variables. We did not compare the proposed approach of handling categorical exposure variable with the one used in Wang et al. [18] because their approach is limited to binary exposure only.

We demonstrated the performance of proposed overall measures with simulation studies for the mediation model with a binary outcome or a continuous outcome and a right-censored mediator. Note that such measures are general and robust and can be employed regardless of whether the outcome variable is continuous or binary and the mediator is censored or not. We also investigated the performance of the proposed overall measures for mediation models in the presence of covariates using simulations (online S5 Table). In particular, we considered a mediation model with a binary outcome. Without loss of generality, we fixed the MAF at 0.3, the censoring percentage at ~20% and the disease prevalence at ~10%. We followed the same procedure as described in the Simulation section to generate data. In addition to the exposure, mediator and outcome, we generated a continuous covariate *Z*~Normal(0, 0.5^2^), which was associated with both the mediator *T* and outcome *Y*. Based on the simulation results, we observed accurate estimations for all the coefficients, as well as *IE* and *PM*. For example, for the scenario 4 in S5 Table, the estimates of *IE* and *PM* were 0.0223 and 0.3000, respectively, which were close to the theoretical values of 0.022 and 0.309. The corresponding coverage probabilities were 0.939 and 0.944, respectively, which were close to the nominal value of 0.95. These results show that the proposed measures are robust even in presence of covariates.

Furthermore, in practice, one may encounter censored data for both outcome variable (e.g., time to onset of disease) and mediator. The approach, using the semiparametric AFT model combined with a pseudo-likelihood function, can be extended to such a mediation model where the outcome variable is also censored. Particularly, one can revise the pseudo-likelihood function to accommodate the survival component. Survival regression models, such as the commonly used Weibull regression model [62], may be employed to address this issue. However, the development of such extension is not straightforward and will need further investigation.

We applied the overall measures of *IE*, *DE*, and *TE* to the motivation study of genetic variants, a woman’s age at menopause, and type 2 diabetes risk. Assuming the additive genetic model for the genetic variants, we identified three variants, rs12744291, rs2503182, and rs11771343, to be included in the mediation analysis because they were associated with both the mediator (i.e., a woman’s age at menopause) and the outcome (i.e., type 2 diabetes status). The results from the mediation analysis showed that a woman’s age at menopause had no mediating effect on the effect of the three genetic variants on type 2 diabetes risk.

Important assumptions required for the mediation analysis have been discussed previously [17,18]. The sensitivity analysis for the assumptions about unmeasured confounders for the derivations of *IE* and *DE* have been extensively conducted and discussed for the motivation study in our previous study [18]. In addition to the “no-unmeasured-confounder” assumptions, we assumed that the mediation model was accurately specified and there were no measurement errors for all the variables in the mediation model. Specifically, for our real data application, we conceptualized the mediation model based on the literature, including the causal orders and causal directions [25–35], and assumed that all the variables, including the exposure, mediator, outcome, and covariates, had no measurement errors.

Besides the assumptions for the mediation analysis, for the parameter estimation approach using the semiparametric AFT model, we assumed that the censoring process for the mediator was independent of the mediator *T*, exposure *X*, outcome *Y*, and covariates *Z*. Sensitivity analysis was conducted previously and showed some degree of robustness for the approach to the violation of the independence assumption [18]. We used the AFT model to relate the exposure to the mediator because it provides the change in the length of survival time as a function of the effect of the exposure, which has an easy way to interpret in the mediation context [17,18]. Other semiparametric survival models, such as the most popularly used Cox proportional model, could be adapted in the mediation model with a censored mediator. However, such adaptation is not straightforward. For example, the measure of effect for the Cox proportional model is the hazard ratio. In such a case, the mediating effect could be difficult to be interpreted because it is the survival time but not the hazard ratio to be expected to have causal effect on the outcome variable. Using other semiparametric survival model in the mediation analysis is of interest to investigate; however, future work is necessary for the derivation of the *IE*, *DE* and *TE* so that these effects can be appropriately interpreted in the mediation context.

Parametric (linear and logistic regressions) and semiparametric approaches (semiparametric AFT model) were employed in the estimation of coefficients for different paths in the mediation model, which usually rely on certain modeling assumptions in one way or another [63–65]. Alternatively, non-parametric approaches to mediation analysis, which has been received a great deal of attention recently, could be considered [63,66–68]. Extension of the current proposed approach for mediation model, where the mediator is censored and the exposure is categorical, to the non-parametric framework will be worthy of future research.

## Supporting information

S1 TableBinary outcome: Simulation results given the minor allele frequency (MAF) = 0.1.Means and standard errors of estimated coefficients for different paths, indirect effects (*IEs*) and proportions of the total effect mediated (*PMs*); and coverage probabilities of the 95% confidence intervals for the estimations of *IE* and *PM*, obtained based on 500 replicates, each with 2000 cases and 2000 controls, given the minor allele frequency (MAF) = 0.1. Different scenarios were considered based on different values of *a*, *b*_*0*_, *b*, censoring percentage (*CP*), and disease prevalence (prev), with *a*_*0*_ = 6 and $\tilde{c}=0.5$.(XLSX)

S2 TableBinary outcome: Simulation results given the minor allele frequency (MAF) = 0.5.Means and standard errors of estimated coefficients for different paths, indirect effects (*IEs*) and proportions of the total effect mediated (*PMs*); and coverage probabilities of the 95% confidence intervals for the estimations of *IE* and *PM*, obtained based on 500 replicates, each with 500 cases and 500 controls, given the minor allele frequency (MAF) = 0.5. Different scenarios were considered based on different values of *a*, *b*_*0*_, *b*, censoring percentage (*CP*), and disease prevalence (prev), with *a*_*0*_ = 6 and $\tilde{c}=0.5$.(XLSX)

S3 TableContinuous outcome: Simulation results given the minor allele frequency (MAF) = 0.1.Means and standard errors of estimated coefficients for different paths, indirect effects (*IEs*) and proportions of the total effect mediated (*PMs*); and coverage probabilities of the 95% confidence intervals for the estimations of *IE* and *PM*, obtained based on 500 replicates, each with 4000 samples, given the minor allele frequency (MAF) = 0.1. Different scenarios were considered based on different values of *a*, *b*, and censoring percentage (*CP*), with *a*_*0*_ = 6, *b*_*0*_ = 1, and $\tilde{c}=0.5$.(XLSX)

S4 TableContinuous outcome: Simulation results given the minor allele frequency (MAF) = 0.5.Means and standard errors of estimated coefficients for different paths, indirect effects (*IEs*) and proportions of the total effect mediated (*PMs*); and coverage probabilities of the 95% confidence intervals for the estimations of *IE* and *PM*, obtained based on 500 replicates, each with 1000 samples, given the minor allele frequency (MAF) = 0.5. Different scenarios were considered based on different values of *a*, *b*, and censoring percentage (*CP*), with *a*_*0*_ = 6, *b*_*0*_ = 1, and $\tilde{c}=0.5$.(XLSX)

S5 TableBinary outcome: Simulation results for a mediation model in presence of a covariate.Means and standard errors of estimated coefficients for different paths, indirect effects (*IEs*) and proportions of the total effect mediated (*PMs*); and coverage probabilities of the 95% confidence intervals for the estimations of *IE* and *PM*, obtained based on 500 replicates, each with 1000 cases and 1000 controls, given the minor allele frequency (MAF) = 0.3. Different scenarios were considered based on different values of *a*, *b*_*0*_, and *b*, with *a*_*0*_ = 6, $\tilde{c}=0.5$, $\gamma_{1}={\tilde{\gamma }}_{1}=0.4$, censoring percentage (*CP*) of ~20%, and disease prevalence (prev) of ~10%.(XLSX)

S1 Appendix Derivations of indirect, direct and total effects. (DOCX)
