# Supplementary material for: Mediation model with a categorical exposure and a censored mediator with application to a genetic study
Source: PLoS One. 2021 Oct 12;16(10):e0257628. doi: 10.1371/journal.pone.0257628 (PMC8509986; doi:10.1371/journal.pone.0257628)
Supplement: S1 Appendix — (DOCX) [file pone.0257628.s006.docx]

### S1 Appendix. Derivations of indirect, direct and total effects.

### In this online supplement, we present the definitions for the indirect effect (*IE*), direct effect (*DE*) and total effect (*TE*) based on the counterfactual framework, as well as the associated assumptions related to unmeasured confounders and consistency required to identify *IE*, *DE* and *TE* in the mediation analysis using the observed data.

Consider a mediation model with a categorical exposure *X* with *k* categories, a mediator *T* subject to right censoring, a continuous outcome *Y* and other covariates *Z*. For the categorical exposure, we create *k*-1 dummy variables, $X_{j}$, $j=1,\ldots,k-1$, where $X_{j}=1$ if $X=d_{j}$, and $X_{j}=0$ if $X\neq d_{j}$. That is, $X=d_{j}$ means $(X_{1}=0, \ldots X_{j}=1, \ldots X_{k-1}=0)$. For the reference category, $X=d_{0}$ is equivalent to $(X_{1}=0, \ldots X_{j}=0, \ldots X_{k-1}=0)$. Let $Y_{d_{j}}$ and $T_{d_{j}}$ be the values of the outcome *Y* and mediator *T*, respectively, that would have been observed had the exposure $X$ been set to $d_{j}$. Let $Y_{d_{j}t}$ be the value of the outcome that would have been observed had *T* and $X$ been set to $t$ and $d_{j}$ respectively. Based on the counterfactual framework, the *IE* compares the expected effects of the mediator *T* at values of $T_{d_{j}}$ and $T_{d_{0}}$ on the outcome *Y* when the exposure $X$ is set to $d_{j}$, conditional on the covariates *Z*, which is denoted as ${IE}_{j\_versus\_0}$ for $j=1,\ldots,k-1$, and defined as follows [[1](#_ENREF_1), [2](#_ENREF_2)]:

$${IE}_{j\_versus\_0}=E \left( Y_{d_{j}T_{d_{j}}} | Z \right)-E\left( Y_{d_{j}T_{d_{0}}} | Z \right).$$

The *DE* assesses the expected effect of the exposure $X$ on the outcome variable *Y* by setting the mediator *T* to the value it would have been if the exposure had the reference value of $d_{0}$, conditional on *Z*, which is denoted as ${DE}_{j\_versus\_0}$ for $j=1,\ldots,k-1$, and defined as follows:

$${DE}_{j\_versus\_0}=E \left( Y_{d_{j}T_{d_{0}}} | Z \right)-E\left( Y_{d_{0}T_{d_{0}}} | Z \right).$$

The *TE* compares the expectations of *Y* if the exposure $X$ had been set to $d_{j}$ versus $d_{0}$, conditional on *Z*, which is denoted as ${TE}_{j\_versus\_0}$ for $j=1,\ldots,k-1$, and defined as follows:

$${TE}_{j\_versus\_0}=E \left( Y_{d_{j}} | Z \right)-E\left( Y_{d_{0}} | Z \right).$$

The following assumptions are required for the derivations of the ${IE}_{j\_versus\_0}$ and ${DE}_{j\_versus\_0}$ [[1-8](#_ENREF_1)]:

(A1) there is no unmeasured confounder for the $X$-*Y* relation conditional on *Z*: $X\perp Y_{d_{j}t} | Z$;

(A2) there is no unmeasured confounder for the *T*-*Y* relation conditional on $X$ and *Z*: $T\perp Y_{d_{j}t} | X, Z$;

(A3) there is no unmeasured confounder for the $X$-*T* relation conditional on *Z*: $X\perp T_{d_{j}} | Z$;

(A4) there is no unmeasured confounder that is affected by $X$ and which itself affects the *T*-*Y* relation conditional on Z: $T_{d_{0}}\perp Y_{d_{j}t} | Z$;

(A5) consistency: $T_{d_{j}}=T$ when $X$ = $d_{j}$ and $Y_{d_{j}t}=Y$ when $X$ = $d_{j}$ and *T* = *t*.

Assuming the assumptions hold, we can assess the conditional expectation $E \left( Y_{d_{j}T_{d_{j}}} | Z \right)$ as shown below.

$$E \left( Y_{d_{j}T_{d_{j}}} | Z \right)=\int E\left( Y_{d_{j}t} | Z, T_{d_{j}}=t \right)\Pr\left( T_{d_{j}}=t | Z \right)dt$$

$$=\int E\left( Y_{d_{j}t} | Z \right)\Pr\left( T_{d_{j}}=t | Z \right)dt by assumption A4$$

$$=\int E\left( Y_{d_{j}t} | Z, X=d_{j} \right)\Pr\left( T_{d_{j}}=t | Z \right)dt by assumption A1$$

$$=\int E\left( Y_{d_{j}t} | Z,X_{1}=0,\ldots,X_{j}=1,\ldots,X_{k-1}=0 \right)\Pr\left( T_{d_{j}}=t | Z \right)dt$$

$$=\int E\left( Y_{d_{j}t} | Z, X_{1}=0,\ldots,X_{j}=1,\ldots,X_{k-1}=0, T=t \right)\Pr\left( T_{d_{j}}=t | Z \right)dt by assumption A2 and A4$$

$$=\int E\left( Y | Z, X_{1}=0,\ldots,X_{j}=1,\ldots,X_{k-1}=0, T=t \right)\Pr\left( T=t | Z, X=d_{j} \right)dt by assumption A3 and A5$$

$$=\int E\left( Y | Z, X_{1}=0,\ldots,X_{j}=1,\ldots,X_{k-1}=0, T=t \right)\Pr\left( T=t | Z, X_{1}=0,\ldots,X_{j}=1,\ldots,X_{k-1}=0 \right)dt$$

$$=\int E\left( Y | t, X_{1}=0,\ldots,X_{j}=1,\ldots,X_{k-1}=0, z \right)dF\left( t \right|X_{1}=0,\ldots,X_{j}=1,\ldots,X_{k-1}=0, z)$$

where $F\left( t \right|X_{1}=0,\ldots,X_{j}=1,\ldots,X_{k-1}=0, z)$is the cumulative distribution function of the mediator given the exposure $X=d_{j}$ and the covariates *Z*.

The conditional expectations $E \left( Y_{d_{j}T_{d_{0}}} | Z \right)$ and $E \left( Y_{d_{0}T_{d_{0}}} | Z \right)$ can be derived similarly:

$$E \left( Y_{d_{j}T_{d_{0}}} | Z \right)=\int E\left( Y_{d_{j}t} | Z, T_{d_{0}}=t \right)\Pr\left( T_{d_{0}}=t | Z \right)dt$$

$$=\int E\left( Y | t, X=d_{j}, z \right)dF\left( t \right|X=d_{0}, z)$$

$$=\int E\left( Y | t, X_{1}=0,\ldots,X_{j}=1,\ldots,X_{k-1}=0, z \right)dF\left( t \right|X_{1}=0,\ldots,X_{j}=0,\ldots,X_{k-1}=0, z),$$

$$E \left( Y_{d_{0}T_{d_{0}}} | Z \right)=\int E\left( Y_{d_{0}t} | Z, T_{d_{0}}=t \right)\Pr\left( T_{d_{0}}=t | Z \right)dt$$

$$=\int E\left( Y | t, X=d_{0}, z \right)dF\left( t \right| X=d_{0}, z)$$

$$=\int E\left( Y | t, X_{1}=0,\ldots,X_{j}=0,\ldots,X_{k-1}=0, z \right)dF\left( t \right| X_{1}=0,\ldots,X_{j}=0,\ldots,X_{k-1}=0, z).$$

Therefore, given the conditional expectations, the ${IE}_{j\_versus\_0}$, ${DE}_{j\_versus\_0}$ and ${TE}_{j\_versus\_0}$ can be assessed as

$${IE}_{j\_versus\_0}=E \left( Y_{d_{j}T_{d_{j}}} | Z \right)-E\left( Y_{d_{j}T_{d_{0}}} | Z \right)$$

$$=\int E\left( Y | t, X_{1}=0,\ldots,X_{j}=1,\ldots,X_{k-1}=0, z \right)dF\left( t \right|X_{1}=0,\ldots,X_{j}=1,\ldots,X_{k-1}=0, z)$$

$$- \int E\left( Y | t, X_{1}=0,\ldots,X_{j}=1,\ldots,X_{k-1}=0, z \right)dF\left( t \right|X_{1}=0,\ldots,X_{j}=0,\ldots,X_{k-1}=0, z),$$

$${DE}_{j\_versus\_0}=E \left( Y_{d_{j}T_{d_{0}}} | Z \right)-E\left( Y_{d_{0}T_{d_{0}}} | Z \right)$$

$$=\int E\left( Y | t, X_{1}=0,\ldots,X_{j}=1,\ldots,X_{k-1}=0, z \right)dF\left( t \right|X_{1}=0,\ldots,X_{j}=0,\ldots,X_{k-1}=0, z)$$

$$-\int E\left( Y | t, X_{1}=0,\ldots,X_{j}=0,\ldots,X_{k-1}=0, z \right)dF\left( t \right| X_{1}=0,\ldots,X_{j}=0,\ldots,X_{k-1}=0, z),$$

$${TE}_{j\_versus\_0}=E \left( Y_{d_{j}} | Z \right)-E\left( Y_{d_{0}} | Z \right)$$

$$=\int E\left( Y | t, X_{1}=0,\ldots,X_{j}=1,\ldots,X_{k-1}=0, z \right)dF\left( t \right|X_{1}=0,\ldots,X_{j}=1,\ldots,X_{k-1}=0, z)$$

$$-\int E\left( Y | t, X_{1}=0,\ldots,X_{j}=0,\ldots,X_{k-1}=0, z \right)dF\left( t \right| X_{1}=0,\ldots,X_{j}=0,\ldots,X_{k-1}=0, z).$$

In our study, we denote *m* as the observed value for the mediator *T* which is subject to right censoring, and $x_{j}$ as the observed value for dummy-coded exposure variable $X_{j}$, $j=1,\ldots,k-1$. The ${IE}_{j\_versus\_0}$, ${DE}_{j\_versus\_0}$ and ${TE}_{j\_versus\_0}$, comparing $X=d_{j}$ with $X=d_{0}$, can be assessed as follow:

$$\begin{matrix} {IE}_{j\_versus\_0} & = & \sum_{m} E \left( Y | m+a_{0}+a_{j}+\tilde{\gamma}z, x_{j}=1, z \right)\eta_{\theta}\left( m | x_{j}=1, z \right) \\ & & -\sum_{m} E \left( Y | m+a_{0}+\tilde{\gamma}z, x_{j}=1, z \right)\eta_{\theta}\left( m | x_{j}=0, z \right) \end{matrix}$$

$$\begin{matrix} & = & \sum_{m} \left\{ b_{0}+b\left( m+a_{0}+a_{j}+\tilde{\gamma}z \right)+\tilde{c}_{j}+\gamma z \right\} \eta_{\theta}\left( m | x_{j}=1, z \right) \\ & & -\sum_{m} \left\{ b_{0}+b\left( m+a_{0}+\tilde{\gamma}z \right)+\tilde{c}_{j}+\gamma z \right\}\eta_{\theta}\left( m | x_{j}=0, z \right) \end{matrix},$$

$$\begin{matrix} {DE}_{j\_versus\_0} & = & \sum_{m} E \left( Y | m+a_{0}+\tilde{\gamma}z, x_{j}=1, z \right)\eta_{\theta}\left( m | x_{j}=0, z \right) \\ & & -\sum_{m} E \left( Y | m+a_{0}+\tilde{\gamma}z, x_{j}=0, z \right)\eta_{\theta}\left( m | x_{j}=0, z \right) \end{matrix}$$

$$\begin{matrix} & = & \sum_{m} \left\{ b_{0}+b\left( m+a_{0}+\tilde{\gamma}z \right)+\tilde{c}_{j}+\gamma z \right\} \eta_{\theta}\left( m | x_{j}=0, z \right) \\ & & -\sum_{m} \left\{ b_{0}+b\left( m+a_{0}+\tilde{\gamma}z \right)+\gamma z \right\}\eta_{\theta}\left( m | x_{j}=0, z \right) \end{matrix},$$

and

$$\begin{matrix} {TE}_{j\_versus\_0} & = & \sum_{m} E \left( Y | m+a_{0}+a_{j}+\tilde{\gamma}z, x_{j}=1, z \right)\eta_{\theta}\left( m | x_{j}=1, z \right) \\ & & -\sum_{m} E \left( Y | m+a_{0}+\tilde{\gamma}z, x_{j}=0, z \right)\eta_{\theta}\left( m | x_{j}=0, z \right) \end{matrix}$$

$$\begin{matrix} & = & \sum_{m} \left\{ b_{0}+b\left( m+a_{0}+a_{j}+\tilde{\gamma}z \right)+\tilde{c}_{j}+\gamma z \right\} \eta_{\theta}\left( m | x_{j}=1, z \right) \\ & & -\sum_{m} \left\{ b_{0}+b\left( m+a_{0}+\tilde{\gamma}z \right)+\gamma z \right\}\eta_{\theta}\left( m | x_{j}=0, z \right) \end{matrix},$$

where $\eta_{\theta}(\cdot)$ is the AFT model error distribution, and *b_0_*, *b*, $a_{0},\text{ }a_{j}, \tilde{c}_{j}$, $\gamma$, and $\tilde{\gamma}$ are regression coefficients as described in the manuscript.

References

1. Vanderweele TJ, Vansteelandt S. Odds ratios for mediation analysis for a dichotomous outcome. Am J Epidemiol. 2010;172(12):1339-48. doi: 10.1093/aje/kwq332. PubMed PMID: 21036955.

2. Wang J, Ning J, Shete S. Mediation analysis in a case-control study when the mediator is a censored variable. Stat Med. 2019;38(7):1213-29. doi: 10.1002/sim.8028. PubMed PMID: 30421436.

3. Huang YT, Pan WC. Hypothesis test of mediation effect in causal mediation model with high-dimensional continuous mediators. Biometrics. 2016;72(2):402-13. doi: 10.1111/biom.12421. PubMed PMID: 26414245.

4. Huang YT, Yang HI. Causal mediation analysis of survival outcome with multiple mediators. Epidemiology. 2017;28(3):370-8. doi: 10.1097/EDE.0000000000000651. PubMed PMID: 28296661.

5. Lange T, Hansen JV. Direct and indirect effects in a survival context. Epidemiology. 2011;22(4):575-81. doi: 10.1097/EDE.0b013e31821c680c. PubMed PMID: 21552129.

6. Pearl J, editor Direct and indirect effects. Proceedings of the Seventeenth Conference on Uncertainty and Artificial Intelligence; 2001; San Francisco, CA: Morgan Kaufmann; 2001.

7. Robins JM, Greenland S. Identifiability and exchangeability for direct and indirect effects. Epidemiology. 1992;3(2):143-55. PubMed PMID: 1576220.

8. VanderWeele TJ. Causal mediation analysis with survival data. Epidemiology. 2011;22(4):582-5. doi: 10.1097/EDE.0b013e31821db37e. PubMed PMID: 21642779.
